# Genomic surveillance: a potential shortcut for effective Chagas disease management

**DOI:** 10.1590/0074-02760220164

**Published:** 2023-01-20

**Authors:** Sophia Lincoln Cardoso de Azevedo, Marcos Catanho, Ana Carolina Ramos Guimarães, Teca Calcagno Galvão

**Affiliations:** 1 Fundação Oswaldo Cruz-Fiocruz, Instituto Oswaldo Cruz, Laboratório de Genômica Funcional e Bioinformática, Rio de Janeiro, RJ, Brasil; 2 Universidade Federal Fluminense, Niterói, RJ, Brasil; 3 Fundação Oswaldo Cruz-Fiocruz, Instituto Oswaldo Cruz, Rio de Janeiro, RJ, Brasil

**Keywords:** Chagas disease, Trypanosoma cruzi, whole genome sequencing, genetic variation, trypanocidal agents, computational biology

## Abstract

Chagas disease is an enduring public health issue in many Latin American countries, receiving insufficient investment in research and development. Strategies for disease control and management currently lack efficient pharmaceuticals, commercial diagnostic kits with improved sensitivity, and vaccines. Genetic heterogeneity of *Trypanosoma cruzi* is a key aspect for novel drug design since pharmacological technologies rely on the degree of conservation of parasite target proteins. Therefore, there is a need to expand the knowledge regarding parasite genetics which, if fulfilled, could leverage Chagas disease research and development, and improve disease control strategies. The growing capacity of whole-genome sequencing technology and its adoption as disease surveillance routine may be key for solving this long-lasting problem.

Approximately 7 million people worldwide are infected by *Trypanosoma cruzi*, the causative agent of Chagas disease (CD).^1^ The infection is endemic in poor rural regions of Latin American countries, habitats to both the vector and the parasite of CD, though recent studies have shown the emergence of triatomine vectors in urban settings suggesting an expansion of risk areas.^2^ ^,^ ^3^ In such regions, the disease is primarily transmitted orally or through vector exposure, sustaining the chance of outbreaks.^4^ ^,^ ^5^ ^,^ ^6^ ^,^ ^7^ ^,^ ^8^ Adding to that, CD has also become a concern in non-endemic settings (*e.g.*, North American, European, and Asian countries) due to population movement,^9^ ^,^ ^10^ ^,^ ^11^ ^,^ ^12^ and approximately 100,000 infected individuals were estimated to live in nine European countries in 2009.^13^ This represents a risk as these patients may vertically transmit CD to their offspring^14^ ^,^ ^15^ or through blood transfusion to other people^16^ ^,^ ^17^ demanding both individual and community health care assistance.

Brazil, one of the endemic countries for CD, has a universal health care system that covers treatment regimens and national vector control programs that have significantly decreased disease incidence in the last decades.^18^ ^,^ ^19^ However, since most acute cases are asymptomatic or present nonspecific symptoms, and chronic cases do not receive a mandatory notification, CD national surveillance and control strategies are currently insufficient, which is reflected in the number of CD-associated deaths: ~ 4,500/year between 2010 and 2019.^20^ Between 2000 and 2011, CD was the leading cause of death by a single pathogen causing a neglected tropical disease in Brazil.^21^ On the other hand, there were only 5,184 acute cases reported between 2001 and 2018, comprising 288/year on average,^22^ suggesting huge underreporting. In other words, in the absence of systematic diagnosis and effective treatment, patients progress to the chronic phase, in which the most severe manifestations and a significantly reduced chance of treatment success prevail.^23^ ^,^ ^24^ The available chemotherapeutics against parasites that cause CD induce severe adverse effects in patients and have low efficacy in the late stage of infection, hampering treatment adhesion and leading to uncertain prognostic.^25^ ^,^ ^26^

Insufficient investments in treatment and vaccine development are an important issue that contributes to the challenge of overcoming the disease. Between 2009 and 2018, US$ 236.31 million was invested in research and development (R&D) for CD, representing only 0.67% of the investment in neglected diseases in the reference period.^27^ ^,^ ^28^ Almost half of this budget (47%) was applied in basic research, 42.5% in drug development and roughly 10% in vaccines and diagnostics. Importantly, as a disease that affects primarily poor populations, pharmaceutical companies historically have shown a lack of interest in R&D for CD, in which funding is mainly provided by the public sector.^27^ Although having a lower death incidence in comparison to other neglected diseases (*e.g.*, HIV/AIDS, malaria, and tuberculosis), which therefore receive proportionally more investments, CD is a relevant cause of premature death and life-long disability.^29^ This “misconception of mildness” may partially account for the lack of urgency of disease management and the persistence of this harmful and silent medical condition.

Another critical aspect of CD control is that *T. cruzi* shows intense genetic diversity, which may contribute to the observed variability in disease outcome, clinical manifestations and treatment efficacy.^25^ ^,^ ^30^ This genetic diversity has also been hampering the search for new treatments, the development of vaccines, and limiting the efficacy of commercial serological diagnosis kits, since all these pharmaceutical technologies rely on the conservation of parasite target proteins. Hence, a more rational approach to disease control should be taken. In this opinion article, we present some of the challenges in CD management imposed by *T. cruzi* genetic diversity, and hypothesise how whole-genome sequencing (WGS) of clinical samples coupled with national disease surveillance could not only enable optimised control strategies in endemic regions but also fuel scientific research projects that focus on developing pharmaceuticals technologies.

The treatment problem

Pharmacological treatment is essential in CD control and management considering disease endemicity and the constant risk of infection. The therapeutic guidelines currently adopted prescribe the only two drugs that have been available for CD treatment since the 1960s: Benznidazole (Bz) and Nifurtimox (first and second-line drugs, respectively). Their exact mechanism of action is not fully understood, but it is known so far that in *T. cruzi* these nitroheterocyclic prodrugs are activated by a type I nitroreductase enzyme (TcNTR) through the reduction of a nitro group.^31^ This reaction produces molecules capable of binding and causing damage to parasite nucleic acids and essential proteins resulting in a trypanocidal effect. Although effective in the initial phase of infection, Bz and Nifurtimox have a long list of intricate problems that may result from their administration.^24^ First, treatment can last up to 90 days and is often indistinctly associated with severe adverse effects which contribute to reduced treatment adhesion. Also, their efficacy is not fully established in the chronic phase and, for this reason, these drugs are frequently not prescribed in these cases.^24^ ^,^ ^25^ ^,^ ^26^ ^,^ ^32^ Therefore, even 113 years after CD’s first description, we cannot assure cure for patients in the late stage of infection and this represents one of the major flaws in CD control as the current epidemiological profile is primarily composed of patients in such condition.

Drug resistance is another remarkable characteristic of the treatment and different mechanisms are suggested to play a role, including efflux pumps, mutations in the TcNTR gene and others that have not been elucidated so far.^32^ ^,^ ^33^ ^,^ ^34^ ^,^ ^35^ ^,^ ^36^ Some strains of *T. cruzi* naturally differ in their susceptibility to Bz and might even be entirely refractory to it.^37^ Resistant clones are readily obtained by drug selection in the laboratory, usually showing cross-resistance to different nitroheterocyclic molecules.^38^ ^,^ ^39^ ^,^ ^40^ As an example, Mejia et al.^39^ observed that *T. cruzi* GAL61 grown in the laboratory in the presence of Bz lost one of the chromosomes containing the TcNTR I gene and that the second copy of the gene acquired mutations that led to non-synonymous amino acid variations in the protein sequence, including conserved positions. Recombinant proteins expressed from the TcNTR I mutant gene were unable to reduce both Bz and Nifurtimox. The authors suggested that the natural range of Bz susceptibility does not result exclusively from TcNTR I intrinsic sequence diversity. Instead, this variability may have different, so far unknown, underlying mechanisms. Finally, they propose that resistance caused by TcNTR I mutations may be a trait that emerges only under selective pressure.

Similarly, Campos et al.^38^ generated Bz-resistant clones of the *T. cruzi* Y strain after four months under selective drug pressure. They observed that the clones also displayed nonsense mutations in the TcNTR I gene and a genome-wide accumulation of single nucleotide polymorphisms (SNP), both emerging during the selection process. Additionally, these clones exhibited cross-resistance against Nifurtimox, impairment of the DNA repair system, and reduced fitness in mice infections. Further investigations are certainly required to explore whether Bz activity can produce new *T. cruzi* variants during a human infection under a treatment regime, but the *in vitro* results demonstrate different mechanisms through which the parasite could survive despite Bz exposure, highlighting its intrinsic genomic plasticity.

Genomic plasticity: a major obstacle to CD treatment, prevention, and diagnosis

The identification of new drug targets is an intrinsically difficult task as many aspects of this process need to be fine-tuned. An ideal target must be essential for parasite survival, structurally unrelated to proteins from the host, expressed in the infecting forms, and present well-documented information about their biochemical characteristics.^41^ Besides, in CD this challenge is broadened by the uncertainty of choosing targets displaying intraspecific or intrastrain sequence diversity, which can lead to treatment failure. For instance, the *T. cruzi* phylogenetic tree is currently based on seven discrete typing units (DTU, TcI-VI, and TcBat), each comprising strains that share a set of well-defined molecular markers but are not genetically identical.^42^

In addition to this constitutive diversity, the parasite also exhibits genomic plasticity, often presenting high SNP density and structural variations (gene and chromosome copy number variations, insertions, and deletions).^43^ ^,^ ^44^ ^,^ ^45^ ^,^ ^46^ ^,^ ^47^ ^,^ ^48^ Its chromosomal DNA is composed of four main types of sequences. The first one is the conserved syntenic coding regions with a single locus that forms the parasite’s core genome. The second type is comprised by gene families encoded in multiple loci, displaying low sequence conservation such as mucins, trans-sialidases, TcGP63, amastin, TcTASV, mucin-associated surface proteins, and cruzipain.^49^ The *T. cruzi* genome also presents non-coding repetitive sequences that participate in different cellular processes composing roughly half of the entire parasite genome.^44^ Finally, thousands of transcriptionally active pseudogenes are also observed. These are intergenic regions displaying protein-coding features, resembling known functional protein(s), bearing substitution(s) and/or insertion(s)/deletion(s) disrupting their open reading frames, which are remnants of the processes of acquisition and gene loss throughout the parasite’s evolutionary history.^50^ Surface protein sequence diversity enables immune evasion during infection and represent a major challenge in the development of vaccines.^51^ Also, variations in gene copy number and in the size of chromosomes have already been reported,^43^ ^,^ ^52^ including in cells that presumably had a clonal relationship, as they were artificially cultured in mammalian epithelial cells.^53^ Moreover, *T. cruzi* can also engage in sexual reproduction promoting genetic exchange between different genotypes even though natural populations exhibit a clonal structure with intense homozygosity and linkage disequilibrium.^54^ ^,^ ^55^

In this sense, some studies have focused on investigating the sequence diversity of *T. cruzi* proteins relevant for CD management. As an example, the ergosterol biosynthesis pathway, a validated drug target in *T. cruzi*, had 20 of its encoding genes displaying extensive inter-strain polymorphisms.^56^ A total of 975 polymorphic sites were identified in all sequences, 28% of them leading to non-synonymous substitutions, with SNP density between genes varying from 2.68 to 11.39 SNPs for every 100 bp. Insights into potential structural impacts generated by non-synonymous variations indicated that the sterol 14-alpha demethylase (TcCYP51), an enzyme also validated as a drug target in *T. cruzi*, presents a crucial substitution (A117S) adjacent to the conserved residue Y116, which has been proposed to play a role in resistance to azole compounds in other species of microorganisms.^57^ ^,^ ^58^

Likewise, ribose-5-phosphate isomerase (Rpi) has been suggested as a new drug target in *T. cruzi*, and molecules that may work as selective inhibitors were identified by virtual screening.^59^ ^,^ ^60^ ^,^ ^61^ An investigation of the sequence diversity among TcRpi protein sequences available in public databases showed 36 distinct clusters among 277 *T. cruzi* genomes (Azevedo et al., unpublished observations). Although most of the 160 amino acids in TcRpi are conserved, some polymorphic positions were observed immediately adjacent to the catalytic residues suggesting functional impact and the need of further investigation with experimental and computational approaches to address this issue.

Even the efficacy of CD serological diagnosis seems to be affected by sequence diversity, as evaluation of antigens used in commercial kits revealed that two out of seven display limited conservation (less than 80% identity) across 52 strains of different DTUs and countries.^62^ This likely contributes to limited performance, causing false-negative results especially when screening samples from Central and North America, given that the sequences used are mostly of antigens from South America strains. The G-FINDER report on R&D for DC, also points out the need for improved pan-geographic accuracy in diagnostics kits and the capacity to perform an early assessment of treatment response.^63^

Beyond treatment and diagnostics, preventing *T. cruzi* infection through vaccination of vulnerable populations would be the state-of-the-art in disease prevention. Unfortunately, we still lack a vaccine and the parasite’s genetic diversity should also be carefully analysed in its development process as protective immunity may be strain-specific.^51^ ^,^ ^64^ Several studies suggest that the *T. cruzi* Tc24 protein could be used as a vaccine antigen candidate with great potential to treat and prevent cardiac fibrosis due to chronic CD. More recently, the possible impact of polymorphisms in Tc24 on immunogenicity was explored, with encouraging results.^65^ ^,^ ^66^ Using WGS reads from samples of DTU TcI to TcVI, Arnal et al.^67^ observed that the Tc24 sequence presents low levels of polymorphisms, loosely associated with the different lineages and not associated with the geographical location of sampling. Only 35 out of 211 codons were under selective pressure, most of them under purifying selection (deleterious variants that are being eliminated) and a small part under diversifying selection (sequence variants that are increasing in frequency). More importantly, the protein regions predicted as epitopes were conserved, supporting the use of Tc24 as a vaccine antigen. Hence, this example suggests that, besides the significant diversity observed in *T. cruzi*, there is still a chance of finding a well-suited protein for vaccine development.

In conclusion, genome plasticity being a remarkable feature in *T. cruzi* biology, partially accounts for the complexity of CD treatment, as well as many of the hurdles faced in effective diagnosis, drug and vaccine development. The above examples highlight the fact that, through evaluating sequence diversity and its functional impact, it is possible to significantly expand the set of information regarding a protein, enabling the choice of the most suitable pharmaceutical targets, and, most importantly, optimising the R&D process. By investigating the type and extent of selective pressure on the target genes it may also be possible to understand the rate and patterns of emerging mutations even before experimental essays, which could save investments and time. This whole evaluation process can be specially performed in a large-scale approach through *in silico* methods, as sequencing technologies are continually evolving as well as computational robustness.

Whole-genome sequencing: an essential tool on CD control strategies

A coordinated effort on exploring sequence diversity might be crucial to understand its extent and to accumulate the knowledge required to improved disease response, either on the individual or populational level. The incorporation of WGS of clinical samples in national protocols for epidemiological surveillance of CD would provide information not only to enable public health measures but also to plan an optimised treatment design strategy; some authors already proposed the incorporation of WGS based molecular genotyping specifically in TriTryps diseases (Leishmaniasis, CD, human African trypanosomiasis) control programs.^68^

It is suggested that *T. cruzi* WGS of clinical samples would provide crucial data to follow the evolution of an epidemic in time and space, characterise new transmission cycles, find the origin of outbreaks and their profile, detect new variants and sexual recombination events, and find genetic markers for traits of clinical and epidemiological relevance.^68^ Through landscape genomics approaches, the provided data could also help understand how the parasite genome changes due to its interaction with different environments, uncovering gene fluxes dynamics and the emergence of new variants, supporting disease control public health measures.^69^ In the R&D field, WGS of genotypes found in a given geographic region would enable the investigation of selective pressure on genes encoding proteins suggested as potential pharmacological targets, making it possible to select a target with a greater chance of success in a population. Also, it would be feasible to identify essential *T. cruzi* genes encoding functional but not structural analogues to human enzymes, which is crucial in drug design pipelines. The impact of *T. cruzi* genetic diversity on drug development strategies has been pointed out by Zingales et al.,^70^ who emphasises that the choice of parasitic strains for *in vitro* testing can determine the outcome of the screening process, but there is still a lack of possible approaches to broadly overcome such an impact.

The Centre for Genomic Pathogen Surveillance has been conducting WGS of clinical samples for surveillance of human pathogens, such as carbapenem-resistant *Klebsiella pneumoniae*,^71^ *Staphylococcus aureus* ^72^ and, more recently, severe acute respiratory syndrome coronavirus 2 (SARS-CoV-2), specially to track antibiotic resistant strains. In a similar manner, the PulseNet International, a network of research laboratories in 88 countries on different continents, has been performing epidemiological surveillance of food-borne pathogens based on molecular methods. The project has recently implemented WGS into their pipelines and is currently aiming to expand its work to low and middle income countries.^73^ ^,^ ^74^ These initiatives highlight that routinely performed large-scale WGS genotyping for infectious disease samples is a feasible approach and its use has been growing due to advances in sequencing technologies.

However, initiatives focused on *T. cruzi* sequencing and expanding the knowledge on its genome have been receiving insufficient investment and many research projects are unfinished. The *T. cruzi* genome project started in 1994 and the complete sequence of the hybrid lineage CL Brener was finished in 2005, alongside the genomic sequences of *Leishmania major* and *Trypanosoma brucei.* ^75^ In 1997, a database for *T. cruzi* genomic and biological information was made available centralising information from different biological databases.^76^ In 2009, the TcSNP database was created to integrate information on genetic variation of different *T. cruzi* genotypes.^77^ In 2007, Andersson and colleagues built a database for *T. cruzi* repeated genes which could help understand the complexity of parasite genome.^78^ Unfortunately, besides their relevance and usefulness for the research community, all these initiatives were discontinued, with the TriTrypDB^79^ and the NCBI databases^80^ currently being the main sources of *T. cruzi* genetic data.

Whole-genome surveillance based on next-generation sequencing might be a potential answer to the impact of genetic variation in CD treatment development. Is estimated that half of the *T. cruzi* genome is composed of repetitive sequences diffusely distributed. In order to overcome this challenge, genomes have been sequenced using hybrid strategies combining a third-generation technology (usually from Pacific Biosciences) and a second-generation technology (such as Illumina), which have shown significant improvement in the number of contigs and the assembly result.^45^ ^,^ ^81^ ^,^ ^82^ The long-reads provided by PacBio’s Single-Molecule Real-Time sequencing (SMRT) technology allow sequencing of long tandem repeats and repeating sequences found at different loci in the genome, overcoming the risk of merging reads from different regions into a single sequence. However, this technology has low coverage and has higher error rate than first- and second-generation sequencing technologies, reducing its ability to identify SNPs and indels that are especially relevant for drug target selection. Therefore, SMRT technology reads need to be corrected by short reads generated by second-generation technologies that features short reads with high coverage or through other approaches. Short-reads coverage ensures proper identification of SNPs and indels, while long-reads increase the likelihood that genomic structure is reliable.

For instance, *T. cruzi*’s NGS raw sequences and their associated metadata could be deposited on a national server for genomic surveillance information of *T. cruzi*. Based on that data, bioinformaticians could work on target discovery workflows. Initially, data quality analysis should be performed, and short reads mapped against long reads. Both should be concatenated to allow the correction of erroneous base-calling and gaps and the resulting long-reads (which became more accurate) can be assembled. The *de novo* assembly becomes more feasible by using long reads allowing the identification of structural variations that are important markers of recombination events. Finally, genome annotations could be performed using basic local alignment search tool (BLAST) algorithms, since many genes and genomes of *T. cruzi* and other trypanosomatids have already been characterised and are publicly available in biological databases.^52^ ^,^ ^56^ ^,^ ^83^ ^,^ ^84^

WGS provides a wealth of information that can be applied for various purposes. In drug design, it would allow the identification of structurally unrelated functional analogues of human enzymes in *T. cruzi*. These are possible candidates for drug targeting, since the minimising potential off-target effects on the human protein. In addition, WGS data allows the investigation of selective pressure on genes that encode proteins suggested as potential pharmacological targets. Many new targets have already been proposed in the literature, but research on their diversity is still scarce.

To investigate sequence diversity, gene sequences from proteins of pharmacological interest could be retrieved from the samples by mapping their reference sequences against the whole genome data. Next, once the sequences from different samples were retrieved, they could be aligned in a multiple sequence alignment to group the sequences based on their identity and similarity creating gene clusters containing homologous sequences originating from the same genome (paralogous) and or homologous sequences shared between two or more genomes (orthologous), as well as groups containing taxonomically restricted sequences (singletons) (Figure). The final stage of the workflow should aim to explore the sequence diversity within the orthologous groups, in which, more conserved positions are expected between sequences of genes shared between representatives of the same DTU. Finally, the most suitable pharmacological targets should have the highest degree of conservation among the largest number of different DTUs comprising the same orthologous group. By analysing clinical *T. cruzi* samples from different DTUs in a specific geographic region, it is possible to evaluate the gene/protein with the highest chance of assuring treatment success when compared to all the potential targets.

**Figure:**
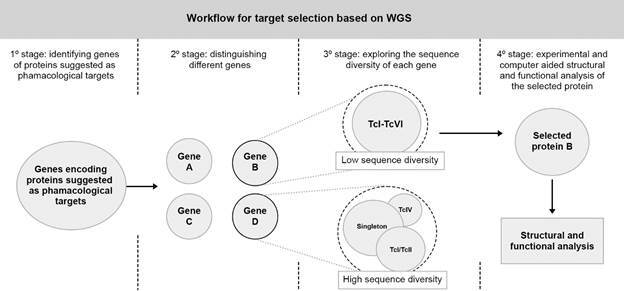
Proposed workflow for investigation of sequence diversity among genes of pharmacological interest to Chagas disease (CD) treatment. In the first stage, gene sequences should be retrieved from whole-genome sequencing (WGS) data by mapping their reference sequences against the genomes. After that, all sequences should be aligned to determine similarity thresholds. Finally, the sequence diversity within each gene cluster could be investigated also using alignment tools but with a more restrictive sequence similarity cut-off.

Although sequencing *T. cruzi* genomes is crucial for CD control, it is important to consider that it still faces technical limitations that must be resolved or improved before its implementation on a large scale. First, reduced parasitemia during chronic infections limits the sensibility of molecular methods due to lower parasitic DNA concentration in blood.^85^ ^,^ ^86^ Having in mind that most patients are asymptomatic and progress to the chronic stage of the disease, this characteristic of the infection may currently represent a limit to the number and types of samples to be sequenced. Another important aspect deals with the maintenance of samples in the laboratory. Different evidence shows that *in vivo* or *in vitro* culture of *T. cruzi* in the laboratory can induce changes in the genetic profile of cells by positively selecting less frequent genotypes that are eventually better adapted to those environments and not necessarily the most prevalent genotype in human infection.^53^ ^,^ ^87^ ^,^ ^88^ Some authors even hypothesise if *T. cruzi*, like other trypanosomatids,^89^ ^,^ ^90^ could be susceptible to aneuploidy induced by environmental stress.^43^ ^,^ ^53^ The genetic profiles observed in sequencing of polyclonal infection samples frequently represent the most abundant infecting genotype but, depending on the abundancy of the different clones, it can result in a mosaic genome that is not representative of any of the real infecting genotypes. To overcome this, improved sample preparation protocols and optimised methods for sequencing data analysis and genome assembly are required.
